# Supplementary material for: Genomic Confirmation of the P-IIIe Subclass of Snake Venom Metalloproteinases and Characterisation of Its First Member, a Disintegrin-Like/Cysteine-Rich Protein
Source: Toxins (Basel). 2022 Mar 23;14(4):232. doi: 10.3390/toxins14040232 (PMC9028321; doi:10.3390/toxins14040232)
Supplement: Supplementary file 1 [file toxins-14-00232-s001.zip › toxins-1629264-supplementary.pdf]

*toxins*

MDPI

*Article*

# Genomic Confirmation of the P-IIIe Subclass of Snake Venom Metalloproteinases and Characterisation of Its First Member, a Disintegrin-Like/Cysteine-Rich Protein

**Kity Požek, Adrijana Leonardi, Jože Pungerčar, Weiqiao Rao, Zijian Gao, Siqi Liu, Andreas Hougaard Laustsen, Alenka Trampuš Bakija, Katarina Reberšek, Helena Podgornik and Igor Križaj**

| | VaaMPIII-2 CDS | | VaaMPIII-3 CDS | | |
|---|---|---|---|---|---|
| | Exon (bp) | Intron (bp) | Exon (bp) | Intron (bp) | |
| Exon 1 | > 52 | | > 52 | | Exon 1 |
| Intron 1 (1) | | 2084 | | 2295 | Intron 1 (1) |
| Exon 2 | 107 | | 107 | | Exon 2 |
| Intron 2 (0) | | 975 | | 883 | Intron 2 (0) |
| Exon 3 | 71 | | 71 | | Exon 3 |
| Intron 3 (2) | | 1835 | | 2309 | Intron 3 (2) |
| Exon 4 | 79 | | 79 | | Exon 4 |
| Intron 4 (0) | | 1111 | | 837 | Intron 4 (0) |
| Exon 5 | 77 | | 77 | | Exon 5 |
| Intron 5 (2) | | 660 | | 4391 | Intron 5 (2) |
| Exon 6 | 196 | | 127 | | Exon 6 |
| Intron 6 (0) | | 2281 | | 1916 | Intron 6 (0) |
| Exon 7 | 57 | | | | |
| Intron 7 (0) | | 655 | | | |
| Exon 8 | 72 | | | | |
| Intron 8 (0) | | 2195 | | | |
| Exon 9 | 170 | | | | |
| Intron 9 (2) | | 1408 | | | |
| Exon 10 | 85 | | | | |
| Intron 10 (0) | | 306 | | | |
| Exon 11 | 128 | | | | |
| Intron 11 (2) | | 2755 | | | |
| Exon 12 | 178 | | | | |
| Intron 12 (0) | | 830 | | | |
| Exon 13 | 90 | | | | |
| Intron 13 (0) | | 1057 | | | |
| Exon 14 | 196 | | 196 | | Exon 7 |
| Intron 14 (1) | | 2307 | | 2619 | Intron 7 (1) |
| Exon 15 | 103 | | 103 | | Exon 8 |
| Intron 15 (2) | | 7931 | | 5870 | Intron 8 (2) |
| Exon 16 | 133 | | 118 | | Exon 9 |
| Intron 16 (0) | | 545 | | 1038 | Intron 9 (0) |
| Exon 17 | > 48 | | > 45 | | Exon 10 |
| **Σ exons** | **1842** | | **975** | | |
| **Σ introns** | | **28935** | | **22158** | |

**Figure S1.** Exon and intron lengths, and intron phases in the VaaMPIII-2 and VaaMPIII-3 genes. Intron phase is shown in parentheses (0, 1 or 2). Intron phases in VaaMPIII-2: 1,0,2,0,2,0,0,0,2,0,2,0,0,1,2,0; and in VaaMPIII-3: 1,0,2,0,2,–,–,–,–,–,–,–,0,1,2,0 (dashes denote missing introns according to VaaMPIII-2).

| N-terminal sequencing | LC-ESI-MS/MS | NCBI accession number |
|---|---|---|
| RAGTECRPARSECDVAEYC | (R)KENDVPIPCAPEDIK(C), (R)KENDVPIPCAPEDIKCGR(L), (K)ENDVPIPCAPEDIKCGR(L), (R)LFCELIKNTCK(Y), (K)YDYSEDPDYGMVDHGTK(C), (K)NTCKYDYSEDPDYGMVDHGTK(C), (R)HCVDVTTAY | MG958499 |

RAGTECRPARSECDVAEYCTGQSAECPTDVFHSNGKPCLNNFGYCYNGNCPIMYHQCYALFG
PNATVGQDGCFEWNKKGESYFYCRKENDVPIPCAPEDIKCGRLFCELIKNTCKYDYSEDPDY
GMVDHGTKCGDGKVCINRHCVDVTTAY

**Figure S2.** Structural identification of the isolated protein as VaaMPIII-3. After reduction and alkylation of Cys residues, one part of the isolated protein was N-terminally sequenced, and the other fragmented with trypsin and subjected to tandem mass spectrometry analysis (LC-ESI-MS/MS). Sequences of seven tryptic peptides were determined. All sequenced parts of the isolated protein are displayed in the table and correspond to VaaMPIII-3 (NCBI accession number: MG958499). On the primary structure of VaaMPIII-3, the identified parts of the sequence (49%) are typed either on black (Edman analysis) or gray background (MS analysis).

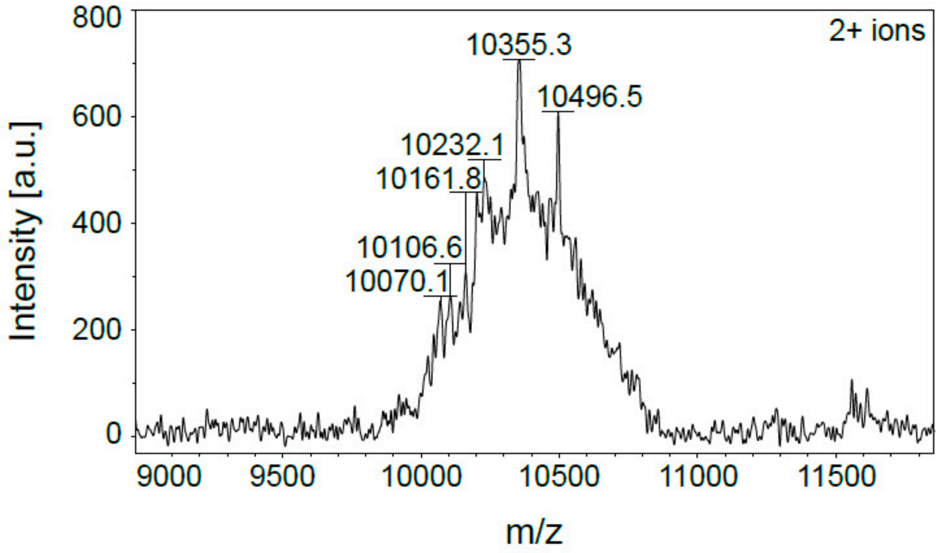


**Figure S3.** Mass spectroscopy of VaaMPIII-3. The VaaMPIII-3 sample was analysed using UltrafleXtreme III MALDI-TOF/TOF mass spectrometer (Bruker, Billerica, MA, USA). Only doubly charged VaaMPIII-3 ions are shown, where the existence of multiple glycoforms of this protein is nicely visible.
